# Brain injury rehabilitation after road trauma in new South Wales, Australia – insights from a data linkage study

**DOI:** 10.1186/s12913-018-3019-8

**Published:** 2018-03-23

**Authors:** Jane Wu, Steven G. Faux, Christopher J. Poulos, Ian Harris

**Affiliations:** 1 St. Vincent’s Hospital, Sacred Heart Rehabilitation Service, 170 Darlinghurst Road, Darlinghurst, Sydney, NSW 2010 Australia; 2 School of Public Health and Community Medicine, University of New South Wales, University Clinics, 9 Judd Ave, Hammondville, NSW 2170 Australia; 3 South Western Sydney Clinical School, UNSW; Whitlam Orthopaedic Research Centre, Ingham Institute for Applied Medical Research, Liverpool Hospital, Locked Bag 7103, Liverpool BC, NSW 1871 Australia

**Keywords:** Traumatic brain injury, Rehabilitation, Data linkage, Road trauma

## Abstract

**Background:**

Population-based patterns of care studies are important for trauma care but conducting them is expensive and resource-intensive. Linkage of routinely collected administrative health data may provide an efficient alternative. The aims of this study are to describe the rehabilitation pathway for trauma survivors and to analyse the brain injury rehabilitation outcomes in the two care settings (specialist brain injury and non-specialist general rehabilitation units).

**Methods:**

This is an observational study using routinely collected registry data (New South Wales Trauma Registry linked with the Australasian Rehabilitation Outcomes Centre Inpatient Dataset). The study cohort includes 268 road trauma patients who were admitted to trauma services between 2009 and 2012 and received inpatient rehabilitation because of a brain injury.

**Results:**

Of those who need inpatient rehabilitation, 62% (*n* = 166) were admitted to specialist units with the remainder (*n* = 102) admitted to non-specialist units. Those admitted to a specialist units were younger (*p* < 0.001), had a lower cognitive FIM score (*p* = 0.003) on admission than those admitted to non-specialist units. Specialist units achieved better overall FIM score improvements from admission to discharge (43 vs 30 points, *p* > 0.001) but at a cost of longer length of stay (median 47 vs 24 days, *p* < 0.001). There were very few discharges to residential aged care facilities from rehabilitation (2% in non-specialist units and none from specialist units). There was a long time lag between trauma and admission to inpatient rehabilitation with only a quarter of the patients admitted to a specialist unit by end of week four. Few older patients (19%) with brain injury were admitted to specialist units.

**Conclusions:**

It is feasible to use routinely collected registry data to monitor inpatient rehabilitation outcomes of trauma care. There were differences in characteristics and outcomes of patients with traumatic brain injury admitted to specialist units compared with non-specialist units.

## Background

The proportion of patients surviving trauma has increased in recent years because of quicker and safer transportation to specialised trauma facilities, more effective emergency care and advances in acute surgical management [1, 2]. Road traumas account for 40% of all trauma admissions. Nearly a third of survivors require inpatient rehabilitation before discharge [1].

About half of road trauma survivors have a traumatic brain injury [2]. While there is a lack of well-designed experimental studies, the available evidence strongly supports the effectiveness of brain injury rehabilitation in order to maximise the patient’s function [3–6].

Current models of brain injury care in New South Wales (NSW), Australia, have been described by the Agency for Clinical Innovation [7]. The elements of the continuum of care are three fold: (1) admission under designated trauma services (usually involving neurosurgery), (2) consultation by rehabilitation services for those requiring inpatient rehabilitation and (3) transfer to a rehabilitation unit when medically stable.

There are two care settings for inpatient brain injury rehabilitation: (1) specialist public brain injury units (operating through the NSW Brain Injury Rehabilitation Program) and (2) non-specialist private and public general rehabilitation units. All residents have free and equal access to specialist units and access is based solely on clinical need and bed availability. However, people of working ages (i.e. below age 65 years) are prioritised for specialist units [8]. The cut off age of 65 years for access to these units is influenced by the Cochrane review [9] on multidisciplinary rehabilitation for acquired brain injury in adults aged 16–65 years. There are no private brain injury units in NSW.

There are differences between the two care settings. Generally, non-specialist units may not offer (or have minimal access to) clinical psychology, may not offer neuropsychology services and may not have ready access to transitional living units. In specialist units, staff caring for patients are provided with specialised training to develop competencies in behavioural change strategies [10]. It can be argued that staff in these specialist units may be more skilled in following behavioural management plans than those in non-specialist rehabilitation units. Patients with brain injury in general rehabilitation units may lack peer support as they are mixed with patients suffering from a variety of conditions.

There are no published data on the proportions of patients admitted into the two care settings in NSW. Patient selection for the two care settings is often decided by a rehabilitation physician or geriatrician. However, non-clinical factors including health system factors (such as bed flow or bed pressures) and insurance status (having a compensable injury or private health cover) can influence this decision [11].

For optimal patient outcomes for those with traumatic spinal cord injury, expert consensus [12] recommends expeditious transfer to a specialist Spinal Cord Injury Unit. These units achieve greater motor FIM gains [13] and better self-reported health outcomes [14] compared to general rehabilitation units. In the absence of comparative outcome data between the two models of care for traumatic brain injury, a recent survey of NSW rehabilitation clinicians has echoed the concern from key stakeholders about whether it is appropriate for patients with brain injury to be sent to non-specialist units [7].

The aims of this study were to investigate the demographic characteristics, define the rehabilitation pathways, and analyse the rehabilitation outcomes after road trauma of a cohort of patients. Prospectively collected trauma registry data were record linked with inpatient rehabilitation data to answer these questions. This is an efficient way of monitoring trauma care as it is less expensive and not as resource-intensive as prospective cohort studies. The state-wide datasets are both population-based so results are generalisable. Analysing the outcomes may also provide insight into any gaps in the delivery of optimal care and identify opportunities for improvement.

## Methods

The study used data from the NSW Trauma Registry which was linked to the Australasian Rehabilitation Outcome Centre (AROC) Inpatient Dataset. AROC collects data from public and private rehabilitation units with almost 100% coverage. NSW Trauma Registry collects data from all trauma centres and it is expected that < 1% of trauma admissions opt out of data collection.

The two datasets were linked using probabilistic methods with the following variables: age, sex, residential postcode, and date of acute discharge = date of admission to rehabilitation. This resulted in a 72% linkage rate with the missed linkages being missed randomly with no obvious bias [15]. Ethics approval was obtained from the NSW Population & Health Services Research Ethics Committee.

**Fig. 1:**
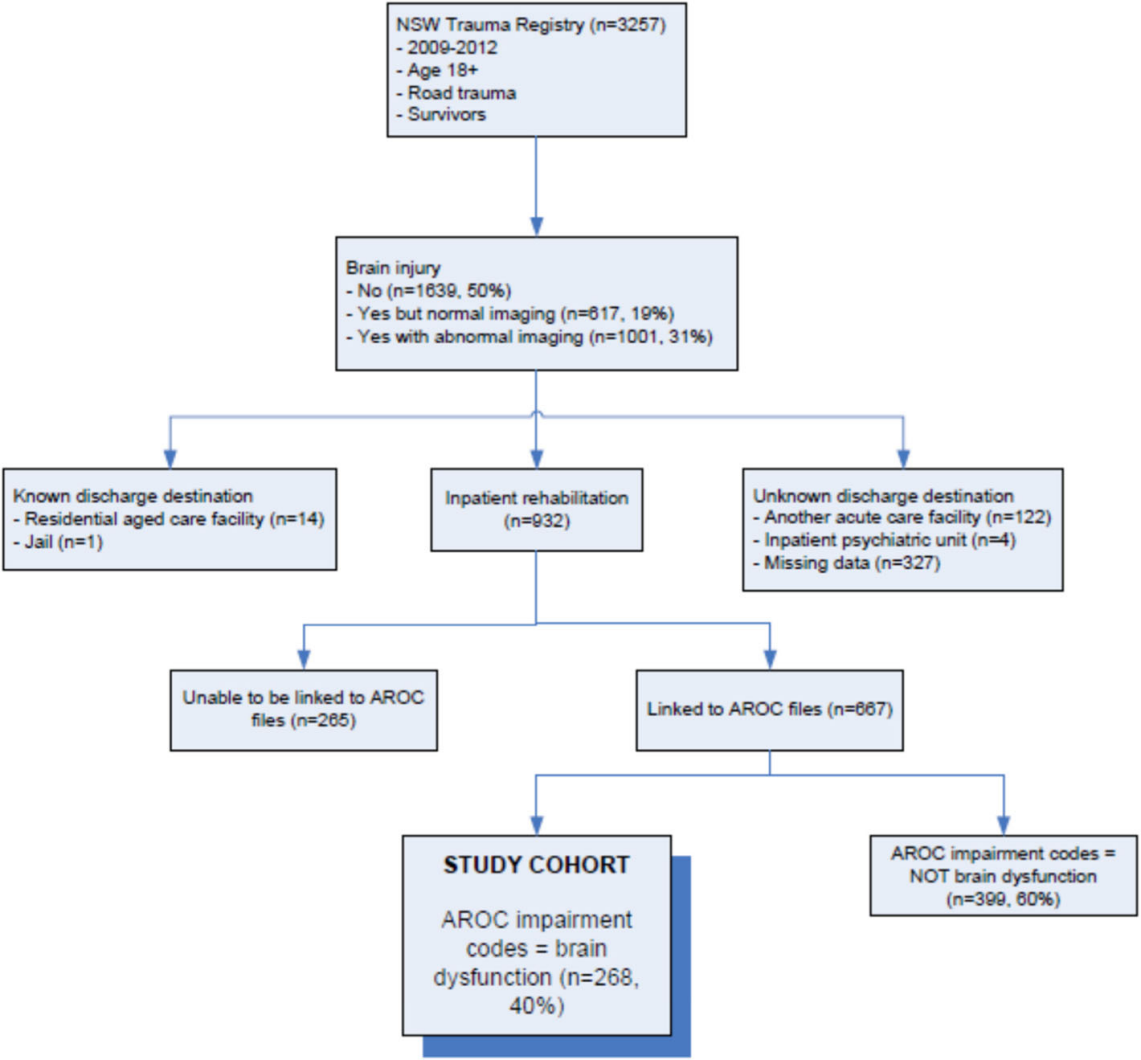
Study cohort extracted from a linked dataset

The cohort for this dataset was all road trauma admissions in NSW for the period 2009–2012 who survived to hospital discharge (Fig. 1). To extract the patients who received brain injury rehabilitation, the following AROC admission impairment codes used were: 2.21 Open traumatic brain injury, 2.22 Closed traumatic brain injury, 14.1 Major multiple trauma (brain + spinal cord injury) and 14.2 Major multiple trauma (brain + multiple fracture/amputation) (Table 1).

**Table 1:** Impairments requiring inpatient rehabilitation

AROC impairment code	Description	Number
2.21	Open traumatic brain injury	22 (8%)
2.22	Closed traumatic brain injury	179 (67%)
14.1	Major multiple trauma (brain + spinal cord injury)	10 (4%)
14.2	Major multiple trauma (brain + multiple fracture/amputation)	57 (21%)

The FIM instrument [16] was used to evaluate progress during inpatient rehabilitation. The scale assessed performance on 18 tasks related to daily living activities. Each task was scored from 1 to 7 based on the relative amount of assistance needed to complete each task. A score of 1 on a particular task indicated that total assistance was needed and less than 25% of the task could be completed alone, whereas a score of 7 reflected the person’s complete independence in performing a particular task. Staff scoring the FIM were required to be appropriately trained in its use and to sit a credentialing exam every 2 years.

The Injury Severity Score is an anatomical scoring system that provides an overall score for patients with multiple injuries [17]. Each injury was assigned an Abbreviated Injury Scale score that classifies individual injuries according to body regions using a 6-point ordinal severity scale. The Injury Severity Score ranges from 1 to 75 and is defined as the sum of squares of the highest Abbreviated Injury Scale scores in the three most severely injured body regions.

Statistical analyses were performed using SPSS v21 (IBM Corp., Armonk, NY, USA). Categorical variables were analysed using chi-squared test. For continuous variables with normal distribution, the mean (SD) were described and the parametric Student’s t-test used. For variables that were not normally distributed, both the mean (SD) and median (IQR) were described and the non-parametric Mann-Whitney U or Kruskal-Wallis test used. A *p* value of < 0.05 was considered significant. Missing values were not imputed.

## Results

The linked dataset contained records of 667 multi-trauma patients who received inpatient rehabilitation after a road accident. In 268 cases (40%) the reason for rehabilitation (based on AROC admission impairment code) was for brain injury rehabilitation (Table 1). Three quarters of the cohort admitted to inpatient rehabilitation, were specifically admitted for traumatic brain injury as the sole main impairment. The remainder (25%) were admitted to rehabilitation for brain injury as well as other impairments due to multiple injuries.

Of those receiving brain injury rehabilitation, 166 (62%) were admitted to a specialist brain injury rehabilitation unit. The remainder were admitted to non-specialist general rehabilitation units (with 75% in the public sector and 25% in the private sector). Of those who were 65 years and over, only 19% (8 out of 42) were admitted to a specialist unit. Of the patients accepted by specialist units, only 5% were older than 65 years.

The mean age of the study population was 42 years (SD 19.5; range 16 to 92); 70% were men. Two-thirds of the study population (67%) were in employment prior to the accident. The rest were either unemployed (13%) or retired (20%). Nearly all (97%) were living in private residences prior to the accident with the remainder in boarding houses or transitional living units.

Time from trauma hospital admission to inpatient brain injury unit admission was analysed in our cohort. The cumulative percentage of patients admitted inpatient rehabilitation is described in Table 2.

**Table 2:** Cumulative percentage of patients being admitted to inpatient rehabilitation after acute trauma

Weeks to admission	General rehabilitation units (*n* = 102)	Specialist brain injury units (*n* = 166)	Total (*n* = 268)
< 1 week	4%	0	2%
1	6%	0	2%
2	24%	3%	11%
3	32%	15%	21%
4	49%	25%	34%
8	82%	80%	80%
> 8	100%	100%	100%

Rehabilitation outcomes as provided in the AROC dataset for patients in specialist units were compared with those in non-specialist units (Table 3). Those treated in non-specialist units were usually older, had less severe injuries and were functionally and cognitively less impaired at admission to rehabilitation compared with those admitted to specialist units.

**Table 3:** Characteristics and outcomes between specialist brain injury units and non-specialist general rehabilitation units

	General rehabilitation units (*n* = 102)	Specialist brain injury units (*n* = 166)	Total (*n* = 268)	*p* value
Age (years) – mean (SD)	50 (± 29)	37 (± 16)	42 (± 19)	< 0.001
Age group (< 65 years)	68 (67%)	158 (95%)	226 (84%)	< 0.001
Male	65 (64%)	124 (75%)	189 (71%)	0.06
Injury severity score				
- Mean (SD)	29 (± 12)	33 (± 11)	32 (± 11)	0.001
- Median (IQR)	25 (21–36)	33 (26–38)	29 (23–38)	
Intensive care admission	75 (74%)	154 (93%)	229 (85%)	< 0.001
Acute length of stay (days)				
- Mean (SD)	36 (± 25)	44 (± 23)	41 (± 24)	
- Median (IQR)	29 (16–49)	40 (28–54)	36 (23–53)	0.001
Rehabilitation length of stay (days)				
- Mean (SD)	41 (± 58)	88 (± 104)	70 (± 92)	
- Median (IQR)	24 (13–43)	47 (29–104)	39 (18–80)	< 0.001
Rehabilitation interruption	5 (5%)	20 (12%)	25 (9%)	0.05
Admission total FIM – mean (SD)	75 (± 29)	59 (± 36)	65 (± 34)	< 0.001
Admission motor FIM – mean (SD)	52 (± 22)	43 (± 28)	46 (± 26)	0.002
Admission cognitive FIM – mean (SD)	22 (± 10)	16 ± (9)	19 (± 10)	0.003
Discharge total FIM				
- Mean (SD)	105 (± 20)	102 (± 27)	103 (± 24)	
- Median (IQR)	112 (101–117)	112 (99–119)	112 (100–118)	0.49
Discharge motor FIM				
- Mean (SD)	77 (± 16)	77 (± 22)	77 (± 20)	
- Median (IQR)	82 (74–86)	87 (78–90)	84 (76–90)	0.001
Discharge cognitive FIM				
- Mean (SD)	28 (± 7)	25 (± 7)	26 (± 7)	
- Median (IQR)	29 (25–33)	26 (21–30)	27 (22–31)	0.001
FIM change – mean (SD)	30 (23)	43 (32)	38 (29)	< 0.001
FIM efficiency				
- Mean (SD)	1.2 (± 1.1)	0.8 (± 0.7)	1.0 (± 0.9)	
- Median (IQR)	0.9 (0.6–1.5)	0.7 (0.3–1.2)	0.8 (0.4–1.3)	0.001
Known discharge destination (*n* = 238)				0.04
- Private residence	79 (90%)	111 (74%)	190 (80%)	
- Hostel	1 (1%)	0	1 (0.5%)	
- Nursing home	1 (1%)	0	1 (0.5%)	
- Group home	0	8 (5%)	8 (3%)	
- Transitional living unit	7 (8%)	31 (21%)	38 (16%)	
Known discharge destination (*n* = 238)				0.009
- Private residence (home)	79 (90%)	111 (74%)	190 (80%)	
- Not home	9 (10%)	39 (26%)	48 (20%)	

The non-specialist units achieved a higher rate of discharge to private residence (*p* = 0.009) and comparable discharge FIM scores (*p* = 0.49) to those of specialist units. There were no discharges to residential aged care facilities from specialist units. Specialist units achieved significantly greater FIM gain (*p* < 0.001) compared with non-specialist units but at a cost of longer length of stay (*p* < 0.001) and therefore lower FIM change per day of admission (FIM efficiency) (*p* = 0.001).

Using the total FIM score to define physical and functional recovery, 62% achieved good recovery (FIM score 109–126), 24% were left with moderate disability (FIM score 72–108) and 14% with severe disability (FIM score < 72).

## Discussion

As trauma systems mature, it is vital to have rehabilitation data to ensure that processes are efficient, the access is equitable and timely and the rehabilitation is effective. This study has documented brain injury rehabilitation outcomes after road trauma in NSW and compared the discharge outcomes between those who received inpatient brain injury rehabilitation in a specialist unit or a non-specialist unit.

In this cohort there appears to be a trend in NSW of younger and more severely injured patients being admitted to specialist units. The 40% of brain injured patients needing inpatient rehabilitation managed in non-specialist units tend to be older and less cognitively impaired. With this large volume managed in non-specialist units, further research into selection criteria may aid in ensuring triage processes provide access to optimal care and health outcomes for brain injury survivors.

Older survivors of brain injury have the potential to improve, just slower and maybe less well than the younger survivors [18]. In our cohort, only 19% of older survivors were admitted to specialist units as these units prioritised patients under the age of 65 years. When faced with limited access to brain injury units, it is conceivable that rehabilitation physicians and geriatricians may consider discharging confused or wandering older brain injury survivors to dementia-specific aged care facilities. This is because general rehabilitation units (which are not usually locked) are often not suitable for this group of patients who are at risk of absconding. Dementia-specific aged care facilities, although secure, lack specialised rehabilitation and surveillance programs. As the population ages, restricting access of older patient to specialist units may need to be reviewed.

The rate of discharge to aged care facilities needs to be explored further. Young people with brain injury, no matter how disabled, should not be discharged to aged care facilities [19]. Specialist units are achieving this goal but the length of time it takes to discharge the more complex cases probably accounts for their overall longer length of stay.

Only 2% of patients were discharged to aged care facilities after receiving their rehabilitation in non-specialist units. Typical rates of discharge to aged care facilities after inpatient stroke rehabilitation in Australia are around 12–20% [20, 21]. This leads us to question whether the “gate keepers” doing the geriatric rehabilitation assessments were sending cases with borderline rehabilitation potential directly to aged care facilities rather than giving them a “trial of rehabilitation”, therefore creating this selection bias in non-specialist units. There is very little literature on patient selection for rehabilitation [22] but this discrepancy suggests further research is needed to develop evidenced based selection guidelines for this population.

There are no published historical data on the outcomes of inpatient specialist or non-specialist brain injury rehabilitation in NSW for comparison. The only published Australian inpatient rehabilitation case series is from a single centre in Queensland in the 1990s but the quality of the data is questionable [23]. There are published series of inpatient brain injury rehabilitation internationally [24–28] but direct comparison with brain injury programs in other countries can be fraught with error. This is due to differences in the model of care, funding, availability of outpatient services and selection bias. The data from this study may be useful for benchmarking purposes and as historical data should there be system wide changes introduced in the future to enhance traumatic brain injury outcomes.

The FIM score has been criticised for being a poor measure of brain injury rehabilitation programs because it is a measure that is dominated by physical disability and only offers a crude assessment for cognitive and psychosocial disability [29]. The UK Rehabilitation Outcomes Collective, a UK national database for specialist rehabilitation in patients with complex disabilities [30], collects UK Functional Assessment Measure (UK FIM + FAM) [31] for this population. The FAM does not stand alone, but extends the 18-item FIM, by adding 12 items that focus on cognitive and psychosocial function. This may be a useful addition to the outcome measures currently collected by AROC.

As trauma systems mature, it is important that the increase in survival comes with no rise in severe disabilities. We believe the discharge FIM score is particularly useful for this purpose. Using a FIM score < 72 to describe severe disability, the 14% in the specialist units that were left with severe disability after inpatient rehabilitation is comparable to a cohort from Norway which was reported as 15% [24]. This measure, if compared over time, may be a useful marker of the non-fatal injury burden.

This study has shown that only one quarter of the patients are transferred to a specialist unit by end of week 4 after injury. The mean length of stay in acute care for those managed in specialist units was 44 (± 23) days. This is at least 2 weeks more than international large published cohorts managed in brain injury units from United States (20–21 days) [25, 26] and Norway (27 days) [24]. It is also longer than the average length of stay (29.4 days) collected by AROC in 2014 from all 19 Australian specialist brain injury units [32]. Although international comparisons need to be interpreted with caution, such difference demands further scrutiny to determine if there are unnecessary delays in NSW. Delays in discharge to brain injury units have been demonstrated to be associated with poorer outcomes [33]. A trial period of increased number of specialist brain injury beds may be worth investigating to see if this can reduce the time from trauma to rehabilitation admission.

As patients in NSW with severe brain injury are spending an average of 6 weeks in acute care, it is important to review the amount of rehabilitation received during this period. Like stroke, recovery in brain injury is fastest in the initial few weeks [34]. Stroke patients typically receive intensive multidisciplinary rehabilitation as “standard care” while being in acute stroke units [35]. However, there is currently no mandate in NSW for the minimum number of allied health therapy hours required in the brain injury population. Intuitively, the two conditions can be considered to be similar from a neuro-rehabilitation point of view. Neuro-rehabilitation improves impairment through neural plasticity [36] so early high intensity therapy may lead to better patient outcomes.

There are two innovative models of care that can be developed to deliver high intensity coordinated rehabilitation to this population group. The first is to use existing in-reach rehabilitation teams that are available in most NSW trauma hospitals [37]. These teams are mobile, going into different acute wards to treat patients who can tolerate increased amounts of therapy and have achievable functional goals. This model of care may be appropriate for trauma hospitals with a smaller volume of brain injured patients.

An alternative model is to set up “early brain injury units” or “hyper-acute rehabilitation” in designated trauma hospitals. These units are a collaborative model of care between intensive care and neurosurgery and rehabilitation medicine. These units can care for patients with severe brain injury who are still requiring high levels of medical care but are adequately resourced to provide intensive rehabilitation until they are stable enough to be transferred to a rehabilitation unit. There are examples of this model of care in Norway [38], Sweden [39], Denmark [40] and England [41] with some preliminary but encouraging results. This model of care may be appropriate for trauma hospitals with a larger volume of brain injured patients.

The strength of this study is that the two linked datasets are population-based, capturing nearly all patients admitted with traumatic brain injury. This minimises the possibility of selection factors having unintended consequences on the study results. This study has also provided a methodology for evaluating the impact of any changes to the NSW brain injury rehabilitation system in the future.

A limitation of this data linkage study is that patients were not matched for severity of brain injury and as such cannot be used to compare relative effectiveness of the two care settings. Markers of severity such as Glasgow Coma Score and duration of post-traumatic amnesia were not available for analysis.

Another limitation is that the study only provides inpatient rehabilitation outcomes and does not provide any data on outpatient rehabilitation outcomes which may be equally important as rehabilitation interventions in this population group extends over time and beyond hospital care to where the person lives and works. In the future, it may be feasible for AROC to conduct phone interviews at certain time points (for examples 6, 12 and 24 months post-injury) to track this group of patients to obtain better data on recovery. A similar model to this has been used by the Victorian State Trauma Registry and they have concluded that the cost of collecting such data is low (10 min required per patient to collect outcome measures) and the follow-up rate was high [42].

This study may have identified opportunities for improvement in the service processes and clinical outcomes for brain injury patients in the future and these include: reviewing the appropriateness of limiting access to specialist units based on age; funding more specialist rehabilitation beds for a trial period to see if this may lead to more timely access; and development of innovative models of care for delivery early neuro-rehabilitation in trauma hospitals.

## Conclusions

This study has demonstrated that routinely collected registry data may be used to monitor trauma rehabilitation outcomes. There are differences in characteristics and outcomes of patients admitted to specialist compared with non-specialist brain injury units. The trend in NSW is that younger and more severely injured patients are being managed in specialist units with the older, less cognitively impaired patients managed in non-specialist units. This study may have identified opportunities for improvement in the service processes and clinical outcomes for brain injury patients in NSW.

## Abbreviations

AROC: Australasian Rehabilitation Outcome Centre
FIM: Functional Independence Measure
IQR: Interquartile range
NSW: New South Wales
SD: Standard deviation
